# Natural Product-Derived Phytochemicals for Influenza A Virus (H1N1) Prevention and Treatment

**DOI:** 10.3390/molecules29102371

**Published:** 2024-05-17

**Authors:** Ruichen Li, Qianru Han, Xiaokun Li, Xinguang Liu, Weijie Jiao

**Affiliations:** 1 College of Pharmacy, Henan University of Chinese Medicine, Zhengzhou 450003, China; rui981220@163.com (R.L.); li96052122@126.com (X.L.); 2 Foreign Language Education Department, Zhengzhou Shuqing Medical College, Zhengzhou 450064, China; dym_jiao@163.com; 3 Co-Construction Collaborative Innovation Center for Chinese Medicine and Respiratory Diseases by Henan & Education Ministry of China, Zhengzhou 450003, China; 4 Academy of Chinese Medical Sciences, Henan University of Chinese Medicine, Zhengzhou, 450003, China; 5 Department of Pharmacy, Henan Province Hospital of Traditional Chinese Medicine, Zhengzhou 450046, China

**Keywords:** influenza A, H1N1, natural products, antiviral agents

## Abstract

Influenza A (H1N1) viruses are prone to antigenic mutations and are more variable than other influenza viruses. Therefore, they have caused continuous harm to human public health since the pandemic in 2009 and in recent times. Influenza A (H1N1) can be prevented and treated in various ways, such as direct inhibition of the virus and regulation of human immunity. Among antiviral drugs, the use of natural products in treating influenza has a long history, and natural medicine has been widely considered the focus of development programs for new, safe anti-influenza drugs. In this paper, we focus on influenza A (H1N1) and summarize the natural product-derived phytochemicals for influenza A virus (H1N1) prevention and treatment, including marine natural products, flavonoids, alkaloids, terpenoids and their derivatives, phenols and their derivatives, polysaccharides, and derivatives of natural products for prevention and treatment of influenza A (H1N1) virus. We further discuss the toxicity and antiviral mechanism against influenza A (H1N1) as well as the druggability of natural products. We hope that this review will facilitate the study of the role of natural products against influenza A (H1N1) activity and provide a promising alternative for further anti-influenza A drug development.

## 1. Introduction

Influenza is a viral respiratory infection that causes acute febrile illness with associated myalgia, headache, and cough. It can result in significantly increased morbidity and mortality during an epidemic [1]. Highly pathogenic strains of influenza A virus have emerged unpredictably but repeatedly in recent history. Since 1918, the year of the “Spanish” influenza outbreak, this virus has caused 50 million deaths in this century [2]. Since then, the introduction of influenza A viruses from poultry or swine herds has led to four world pandemics, all causing high mortality, global health threats, and large economic losses [3,4]. Due to the rapid variation of influenza and the low vaccine penetration rate, antiviral drug therapy remains the core treatment for influenza. Anti-influenza virus drugs approved by the U.S. Food and Drug Administration (FDA) include M2 inhibitors, adamantanes (rimantadine and amantadine), NA inhibitors (NAI; peramivir, zanamivir, and oseltamivir), and, more recently, the cap-dependent endonuclease inhibitor targeting the PA polymerase subunit (baloxavir) [5,6,7]. However, adverse drug reactions and the emergence of resistant strains of the virus have made the development of safe and effective new antiviral drugs increasingly urgent for both therapeutic or prophylactic purposes.

Natural products refer to compounds or substances derived from natural resources such as animals, plants, microorganisms, minerals, etc., which are not artificially synthesized or modified. [8]. The use of natural medicine has a long history, and it has been widely considered the focus of development programs for new, safe anti-influenza drugs [9]. In this review, we aimed to provide a new perspective for the development of novel anti-H1N1 drugs. We conclude the review of newly discovered natural products with anti-influenza A (H1N1) activity with a call for more researchers to focus on natural products as potential drugs for the treatment of influenza A (H1N1).

## 2. Treatment Strategies against Influenza A (H1N1)

**Figure 1:**
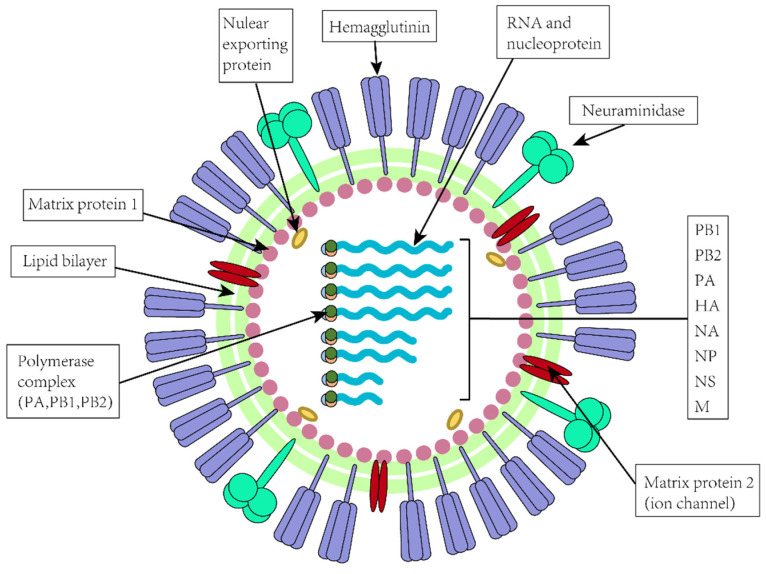
The structure of influenza A virus. PB1, polymerase basic protein 1; PB2, polymerase basic protein 2; PA, polymerase acidic protein; HA, hemagglutinin; NA, neuraminidase; NP, nucleoprotein; PA, polymerase acidic protein; NS, non-structural protein; M, matrix protein.

The influenza virus is a negative, single-stranded RNA virus belonging to the Orthomyxoviridae family [10]. Its genome is about 13.6 kb and consists of eight independent RNA fragments of different sizes, encoding 10 proteins, respectively: HA, NA, PA (RNA polymerase subunit PA), PB1 (RNA polymerase subunit PB1), PB2 (RNA polymerase subunit PB2), nuclear protein (NP), M (membrane proteins including M1 and M2, encoded by the same RNA fragment), and NS (non-structural proteins including N1 and N2, encoded by the same RNA fragment) [11] (Figure 1). According to the NP and membrane protein and their genetic characteristics, influenza virus can be divided into A, B, and C types [11]. Among them, influenza A viruses are more prone to antigenic mutations and are more variable than the other influenza viruses. Therefore, it is not only the most common and lethal type of influenza virus but also the main virus that causes seasonal or pandemic influenza. Furthermore, 18 different hemagglutinin (HA) subtypes (H1–H18) and 11 different neuraminidase (NA) subtypes (N1–N11) of influenza A viruses currently exist, which together define the influenza A virus subtype [12,13,14].

**Figure 2:**
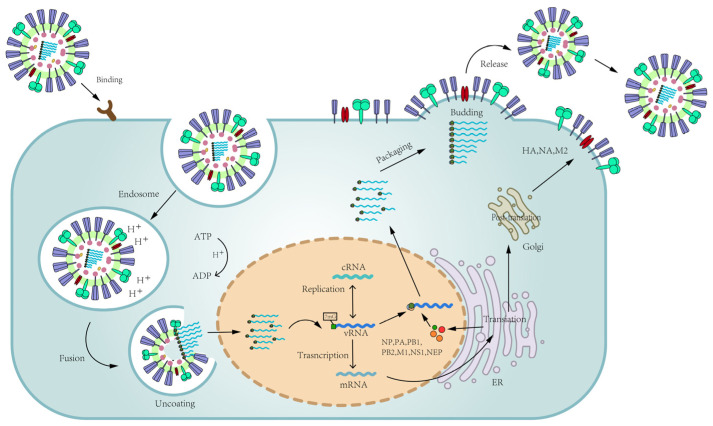
The life cycle of the influenza A virus and the targets of natural compounds against influenza A viruses (H1N1). Blue rounded box, anti-H1N1 compound; red arrow, target. (1) Influenza virus hemagglutinin (HA) binds to sialic acid-presenting receptors on the surface of host cell membranes. Virus particles enter the host cells to form endosomes through receptor-mediated cellular endocytosis. (2) Endosome acidification promotes conformational changes of HA, resulting in the uncoating of the virus and release of the vRNP into the cytosol of the host cell, with further transportation to the nucleus. (3) vRNPs enter the nucleus to initiate the viral mRNA. HA, NA, and M2 are processed at the ER apparatus and Golgi before transport to the cell surface. Influenza virus polymerase can synthesize viral mRNA and vRNA. The vRNA is first converted into plus-strand cRNA; then the new vRNA is synthesized using cRNA as a template. (4) Viral proteins and vRNA are transported to the cell surface to assemble progeny viruses and initiate the virus budding process. The progeny virus is then released from the surface of the infected cells and seeks new host cells to infect. SA, sialic acid; HA, hemagglutinin; NA, neuraminidase; M2, ion channel protein; NP, nucleoprotein; PA, polymerase acidic protein; PB1, polymerase basic protein 1; PB2, influenza polymerase subunit protein; M1, matrix protein1; NS1, non-structural protein 1; NEP, nuclear export protein; vRNP, viral RNA ribonucleoprotein; NS2, non-structural protein 2; ER, endoplasmic reticulum.

The process of influenza virus infection and proliferation mainly includes the following steps. (1) Adsorption: In the process of influenza virus infection, the virus first recognizes and adsorbs to the sialic acid (SA) receptor on the surface of the host cell glycoprotein through its surface HA and then enters the host cell to form intracellular bodies (endosomes) through receptor-mediated endocytosis. (2) Integration: The conformational change of the endosome mediates the fusion of the viral envelope with the endosome membrane and releases the ribonucleoprotein (VRNP) into the cytoplasm of the host cell. (3) Nuclear import and replication: The endosome activates the ion channel M2, which generates an inward proton flow that directs the transport of VRNP into the nucleus for viral RNA replication. Polymerase proteins (PB2, PB1, and PA) and NP play important roles in the transcription and replication of influenza viruses. (4) Assembly and release: The newly generated progeny viral RNA is transported out of the nucleus into the cytoplasm and assembled into the mature virus. The new viral particles are still attached to the outer membrane of the host cell through HA (Figure 2). Thus, blocking any of these four steps could prevent influenza virus infection, and key parts or key proteins of these steps would be possible targets for anti-influenza virus drugs. Antiviral small-molecule compounds currently on the market or in clinical use mainly include specific influenza virus inhibitors (M2 ion channel blockers, NA inhibitors, PA inhibitors, and PB2 inhibition agents) and some broad-spectrum antiviral drugs [15,16,17].

Influenza virus hemagglutinin (HA) binds to sialic acid-presenting receptors on the surface of host cell membranes. Virus particles enter the host cells to form endosomes through receptor-mediated cellular endocytosis. (2) Endosome acidification promotes conformational changes of HA, resulting in the uncoating of the virus and release of the vRNP into the cytosol of the host cell with further transportation to the nucleus. (3) vRNPs enter the nucleus to initiate the viral mRNA. HA, NA, and M2 are processed at the ER apparatus and Golgi before transport to the cell surface. Influenza virus polymerase can synthesize viral mRNA and vRNA. The vRNA is first converted into plus-strand cRNA; then the new vRNA is synthesized using cRNA as template. (4) Viral proteins and vRNA are transported to the cell surface to assemble progeny viruses and initiate the virus budding process. The progeny virus is then released from the surface of the infected cells and seeks new host cells to infect.

### 2.1. M2 Inhibitors

The main function of the M2 ion channel is to transport protons and induce fusion of the viral membrane with the endosomal membrane. M2 ion channel blockers act by binding to the interior of the ion channel, blocking the influx of protons and thereby preventing hemagglutinin-mediated membrane fusion [18]. The first antiviral drugs approved by the FDA to treat influenza A virus infection were adamantanes, including amantadine and rimantadine. They can block the M2 membrane protein ion channel, thereby preventing the dissociation of M1 protein and ribonucleoside protein and further blocking the initiation of the replication process of influenza A virus RNA to achieve therapeutic effect [19,20]. The M2 protein is only present in the membrane protein of influenza A virus. Therefore, these drugs belong to specific inhibitors of influenza A viruses and are ineffective against influenza B viruses. M2 ion channel blockers have been implicated in the virulence occurring in the digestive and autonomic nervous systems [21]. Forty years of long-term, widespread, and extensive use of these drugs has caused most influenza A viruses to be severely resistant to them [22,23].

### 2.2. NA Inhibitors

Neuraminidase (NA), also known as sialidase, plays a key role in the life cycle of the influenza virus. Currently, four drugs have been approved for clinical treatment; they include zanamivir (zanamivir, ZAN; Relenza^®^), oseltamivir phosphate (oseltamivir phosphate; Tamiflu^®^), peramivir (peramivir1; Rapivab^®^), and ranamivir (laninamiviroctanoata; Inavir^®^). In 1974, Palese et al. [24] discovered Neu5Ac2en, a sialic acid-analogue NA inhibitor. Zanamivir was successfully developed [25] in 1991 and approved by the FDA in July 1999. In 1996, oseltamivir was successfully developed and approved by the FDA in October 1999 [26]. Peramivir was approved for marketing in 2009 [27]. Ranimivir octanoate, a prodrug of ranimivir, was marketed in Japan in 2010 [26,27]. Currently, NA inhibitors are the research hotspot for anti-influenza virus drugs. However, existing NA inhibitors are not perfect drugs against influenza A viruses. For example, zanamivir has a high antiviral activity but low bioavailability and is rapidly excreted in the kidneys; oseltamivir often has side effects such as nausea and vomiting in adults [7]. Moreover, due to the widespread clinical application of existing NA inhibitors, influenza viruses mutate and develop different degrees of resistance to these drugs through the change in shape of the NA catalytic position to reduce the sensitivity of the virus to the NA inhibitor [7,28]. The catalytic site of NA consists of eight functional residues, and the surrounding eleven framework residues ensure the stability of the active site structure. Due to the hydrophobic bond of oseltamivir, NA must undergo rearrangement to adapt to drug binding. Any mutation that affects the rearrangement may reduce the binding affinity of oseltamivir [29]. In addition to H275Y mutation, which can enhance the drug resistance of oseltamivir, D199G, S247N, and I223M mutations can reduce the sensitivity of oseltamivir; I223R mutation can reduce the sensitivity of oseltamivir and zanamivir; and Q136K mutation can reduce the sensitivity of zanamivir [30].

### 2.3. Viral Polymerase Complex Inhibitor

Influenza virus polymerase is composed of alkaline PB1, PB2, and polymerase acidic (PA) [31]. The PB2 subunit binds to the cap of the host cell pre-messenger RNA and is subsequently cleaved by cap-dependent endonucleases in the PA subunit. This “cap-capturing” process provides RNA primers for the transcription of viral MRNAs through the RNA-dependent RNA polymerase function of PB1 [32]. These include the PB1 inhibitor, favipiravir (Avigan), which was approved for influenza treatment in Japan in 2014 [33]. Pimodivir, also known as JNJ-63623872 and VX-787, is a PB2 inhibitor with selective activity against influenza A viruses and is suitable for oral administration [34]. VX-787 has performed well in clinical studies. Baloxavirmarboxil (44, S-033188), a PA inhibitor, was marketed in Japan in February 2018 and in the United States in October 2018 as Xofluza for the treatment of influenza A and B, respectively [35].

### 2.4. NP Inhibitors

NP is one of the most abundant viral proteins produced during viral replication. During the viral life cycle, NP participates in the formation of viral ribonucleoprotein complexes (vRNPs) by binding to influenza virus RNA and polymerase subunits (PB 1, PB 2, and PA) and also participates in the nuclear import, replication, and export of vRNPs [36,37]. In recent years, NP has become a hotspot in antiviral drug development because of its important role in virus replication. Naproxen was identified by computer screening as a dual inhibitor of NP and COX2, a type of cyclooxygenase [38]. Naproxen can inhibit viral replication by targeting the RNA groove of NP and preventing NP from interacting with viral RNA. Naproxen showed good antiviral effect against H1N1 and H3N2 viruses both in vivo and in vitro [39]. Due to the strong serum variability of NP antigen, many researchers once thought that NP was not suitable as a drug target against influenza viruses. However, some NP inhibitors that are effective against both influenza A and B viruses have recently emerged. Kao et al. [40] confirmed that NP can serve as a target for influenza treatment drugs by forward chemical genetic technology and found a small-molecule compound named nucleozin, which targets NP. Nucleozin can induce NP aggregation into large NP complexes, completely antagonizing their entry and accumulation in the nucleus and inhibiting virus replication. Animal research results indicate that nucleozin can effectively treat highly pathogenic H5N1 influenza virus-infected mice [40]. Liu et al. [41] found that the groove surface between the head and body domains of influenza A NP are covered by a large number of conserved residues, playing an important role in influenza virus RNA binding. To explore the mechanism of NP binding to RNA, they performed [41] a series of directed induced mutations in the RNA binding slots and characterized the interaction between RNA and NP by surface plasmon resonance (SPR). Liu et al. [41] identified an influenza virus NP inhibitor, (E, E) -1,7-bis (4-hydroxy-3-methoxyphenyl) -1,6-heptadiene-3,5-dione. This inhibitor reduces the RNA binding affinity of NP and hinders viral replication. NP is highly conserved among influenza A virus strains from different species, indicating that influenza viruses are less likely to develop resistance to NP inhibitors. The multiple drug binding sites and high sequence conservation of NP makes it a highly anticipated drug target [42].

## 3. Natural Compounds That Exert Anti-Influenza A Effects

### 3.1. Marine Natural Products

**Figure 3:**
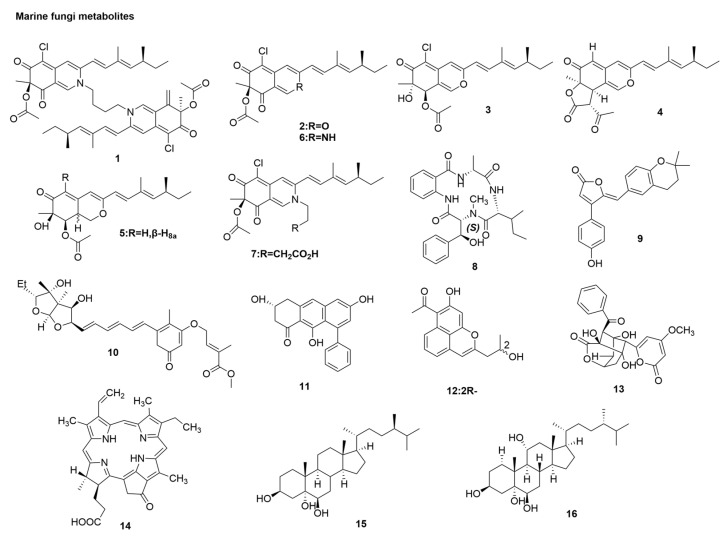
Marine fungi metabolites with activity against H1N1.

The ocean is the largest treasure house of resources on the earth, with a huge diversity of species. Marine natural products have unique chemical structure and function, high biological activity, and research and development potential [43]. As a benefit from the unique aquatic environment and rich species in the sea, the active metabolites produced by marine microorganism are more novel in structure and more unique in function than terrestrial microorganisms [44]. Azaphilones are reported to be a class of fungal metabolites with antibacterial, antiviral, anti-inflammatory, antioxidant, nematocidal, and cytotoxic biological activities [45,46]. Currently, more than 430 kinds of azaphilones have been extracted from marine and terrestrial fungi. Among them, sclerotiorin E, (+) sclerotiorin, TL-1-monoactate, ochrephilone, 8-acetyldechloroisochromophilone III, scleratioramine, and isochromophilone IX (**1**–**7**), isolated form *Penicillium sclerotiorum* [47,48] (Figure 3), showed a comparable protective effect of canine kidney cells from H1N1 infection [49,50] (seeing Table 1). Data has shown that the dimer azaphilones had a stronger antiviral activity than the monomer, and this antiviral activity has no relationship with the substitution of chlorine atoms on C-5 or the oxygen atom connected to C-1 [51]. He et al. isolated a new antiviral cyclic tetrapeptide containing a rare 3-OH-N-CH3-Phe residue named asperterrestide A (**8**) from the fermentation broth of the marine-derived fungus *Aspergillus terreus* SCSGAF0162 [52] (Figure 3). CPE was performed for the influenza virus strains A/WSN/33 (H1N1) and strain A/Hong Kong/8/68(H3N2) to determine the inhibition of viral replication in cellular MDCK [52]. A cytotoxicity assay showed that asperterrestide A exhibited cytotoxicity against human cancer cell lines U937 and MOLT4, with IC_50_ values of 6.4 μM and 6.2 μM, respectively. Asperterrestide A showed inhibitory effects on A/WSN/33 (H1N1) and A/Hong Kong/8/68 (H3N2), with half-maximal inhibitory concentration (IC_50_) values of 15 and 8 μM, respectively (Table 1) [52]. Two new rubrolides were isolated from the fermentation broth of the marine-derived fungus *Aspergillus terreus* OUCMDZ-1925 [52]. Rubrolides S (**9**) (Figure 3) showed comparable or superior anti-influenza A (H1N1) virus activity to that of ribavirin, with an IC_50_ value of 87 µM (Table 1) in CPE inhibition assay [53]. A cytotoxicity assay showed that rubrolides S (**9**) was non-cytotoxic to A549, HL-60, HeLa, and HCT-116 cell lines (IC_50_ = 430 mM). In a study by Tian et al. [54], a new asteltoxin from *Aspergillus* sp. SCSIO XWS02F40, asteltoxin E (**10**) (Figure 3), exhibited an inhibitory activity against H1N1, with an IC_50_ value of 4 µM in a CPE inhibition assay [54] (Table 1). Five new phenolic polyketides and two known compounds were isolated and identified by Zhu et al. [55] from the fermentation broth of *Streptomyces* sp. OUCMDZ-3434 associated with the marine green algae *Enteromorpha prolifera*. The antiviral activity of seven compounds against influenza A H1N1 virus was evaluated using the CPE inhibition assay [49,50]. Wailupemycin J (**11**), R-wailupemycin K (**12**), and 5-deoxyenterocin (**13**) (seeing Figure 3) showed anti-H1N1 virus activity, with 47.8%, 42.5%, and 60.6% inhibitory activities, respectively, at 50 mg/mL (ribavirin, 45.3% inhibition) [55] (Table 1). The cytotoxicity assay of these compounds on HeLa cells showed that none of them exhibited cytotoxicity (IC_50_ > 50 μM). Pyropheophoride a (PPa) (**14**) (Figure 3) is a porphyrin derivative isolated from the seafood *Musculus senhousei* (*M. senhousei*), which exhibits promising anti-influenza activity in vitro. PPa showed inhibitory effects on A/Puerto Rico/8/34(H1N1), with an IC_50_ value of 0.17 μg/mL (Table 1). Studies have shown that PPa may interact with viral envelope lipids. Thus, the fusion of virus and cell membrane was blocked, and anti-H1N1 virus activity was achieved [56]. Antiviral activity of the South China Sea soft coral *Sarcophyton* sp. and all isolated compounds against H1N1 virus by CPE, namely (24R)-methylcholest-7-en-3β,5α,6β-triol (**15**) (Figure 3) and (24S)-ergost-3β,5α,6β, 11α-tetraol (**16**) (Figure 3), showed relatively strong inhibitory effects on A/Puerto Rico/8/34(H1N1), with IC_50_ values of 19.6 and 36.7 μg/mL (Table 1) [57].

### 3.2. Flavonoids

Flavonoids are one of the largest classes of secondary metabolites in plants. They are bio-synthesized through the pathways of shikimic acid/phenylpropane and acetic acid/malonic acid. Flavonoids contain a common C6-C3-C6 skeleton, which has two aromatic rings that are connected by a three-carbon bridge, typically forming a benzene chromone arrangement. According to degree of unsaturation and substitution mode, flavonoids can be classified into sub-classes: flavones, flavonols, flavanone, flavan-3-ol, anthocyanins, dihydroflavonols, and isoflavones as well as the form of biogenetic intermediate chalcone. In recent studies, flavonoids have been widely recognized as NA inhibitors.

**Figure 4:**
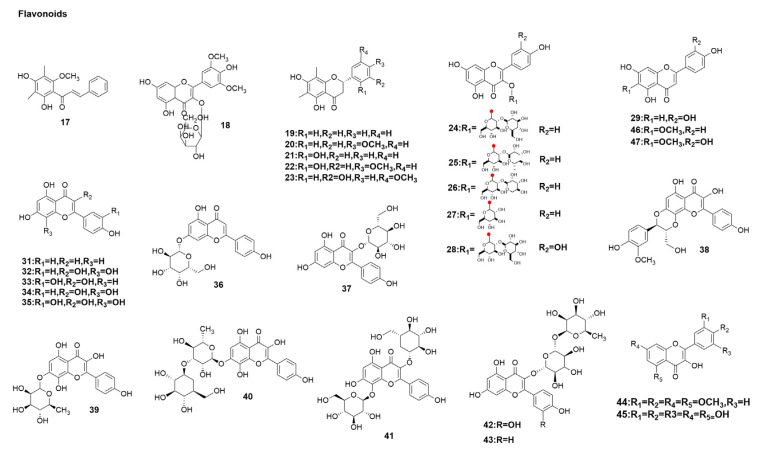
Flavonoids with activity against H1N1.

*Cleistocalyx operculatus* (Roxb.) Merr. and Perry (Myrtaceae), widely used in traditional medicine in China, Vietnam, and other tropical countries, has diverse biological activities [93]. It is utilized to treat various conditions, including fever and bacterial dysentery, and their main chemical components, flavonoids, exhibit antioxidant, anti-hyperglycemic, anti-influenza, and cholinesterase-inhibitory properties [93,94,95,96]. Therefore, to further investigate the inhibitory activity of their extracts against influenza virus NA, Ha et al. [58] repeatedly isolated nine compounds from rhizomes of *Pentarhizidium orientale* through a series of chromatographic procedures. By a CPE inhibition assay, 2′,4′dihydroxy-6′-methoxy-3′,5′-dimethylchalcone (**17**) and 3′,5′-dimethylether 3-O-β-D-galactopyranoside (**18**) (Figure 4) not only had inhibitory activity against the influenza virus H1N1 A/PR/8/34 NA but also showed inhibitory activity against wild-type novel swine influenza (WT) and oseltamivir-resistant (H274Y mutation) viral NA. The IC_50_ values were 8 and 9 μM, respectively (seeing Table 1). The inhibition patterns of 2′,4′dihydroxy-6′-methoxy-3′,5′-dimethylchalcone (**17**) and 3′,5′-dimethylether 3-O-β-D-galactopyranoside (**18**) (see Figure 4) were further investigated using the double-reciprocal Lineweaver–Burk plot, which showed that the two compounds exhibit a noncompetitive inhibition pattern against influenza NA [58].

In one study, C-methylated flavonoids were reported to exhibit anti-influenza virus (H1N1)-inhibitory effects [97]. *Pentarhizidium orientale* (Hook.) Hayata (Onocleaceae) (syn. *Matteuccia orientalis*) is a perennial pteridophyte that is distributed mainly in East Asia and the temperate regions of the northern hemisphere [59,97]. As part of the present investigation on the bioactive compounds from a Korean medicinal plant [98], the phytochemicals in an 80% aqueous MeOH extract of the *P*. *orientale* rhizome were purified [59]. The NA-inhibitory activity of several isolated compounds from *P. orientale* against the H1N1 influenza virus was tested using the CPE inhibition assay [59]. The results indicated that demethoxymatteucinol (**19**), matteucinol (**20**), matteucin (**21**), methoxymatteucin (**22**), and 3′-hydroxy-5′-methoxy-6,8dimethylhuazhongilexone (**23**) (see Figure 4) exhibited NA-inhibitory activities, with IC_50_ values ranging from 24 to 30 μM (Table 1). These compounds were compared with oseltamivir [59]. Demethoxymatteucinol (**19**) exhibited cytotoxicity against MDCK cells (CC_50_, 77.6 μM), while other compounds did not show significant cytotoxicity.

**Figure 5:**
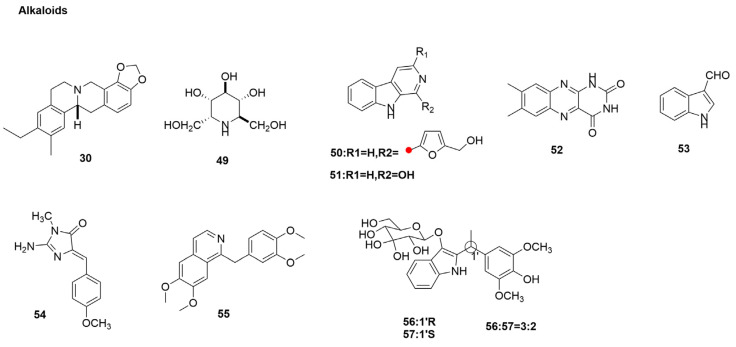
Alkaloids derivatives with activity against H1N1.

Bee pollen is a combination of plant pollen and bee secretions and nectar. Bee pollen contains bioactive compounds including proteins, amino acids, lipids, carbohydrates, minerals, vitamins, and polyphenols [60]. kaempferol-3-sophoroside (**24**), kaempferol-3-neohesperidoside (**25**), kaempferol-3-sambubioside (**26**), kaempferol-3-glucoside (**27**), quercetin-3-sophoroside (**28**), luteolin (**29**) (Figure 4), and chelianthifoline (**30**) (Figure 5) A-inhibitory activities of seven compounds were evaluated against the recombinant influenza viral subtypes, namely H1N1, H3N2, and H5N1, with zanamivir as a positive control. All compounds inhibited NAs in a dose-dependent manner. The IC_50_ values of seven compounds on the NAs from influenza H1N1 ranged from 11 to 101 µM (see Table 1). The modes of inhibition of these compounds were further investigated using Dixon plots, which showed that six compounds other than quercetin-3-sophoroside (**28**) exhibited non-competitive inhibitory activities. The authors further analyzed the structure–activity relationship and concluded that the bulky sugar moiety in compounds **24**–**28** causes a decrease in activity. The cytotoxicity assessment revealed that kaempferol-3-sophoroside (**24**), kaempferol-3-neohesperidoside (**25**), kaempferol-3-sambubioside (**26**), kaempferol-3-glucoside (**27**), quercetin-3-sophoroside (**28**), luteolin (**29**), and chelianthifoline (**30**) exhibited no toxicity to MDCK cells at a concentration of 100 µM, with a cell viability of 100% [60].

*Rhodiola rosea* L. belongs to the plant family Crassulaceae, which is widely distributed in the world. It is a traditional Chinese medicine with various biological activities, such as anti-diabetes, anti-cancer, anti-inflammatory, anti-aging, and anti-depression [99,100,101]. Five flavonols, i.e., kaempferol (**32**), herbacetin (**34**), rhodiolinin (**38**), rhodionin (**39**), and rhodiosin (**40**) (Figure 4), were isolated from *Rhodiola rosea* by Lee et al. [61] and compared with the commercially available flavonoids apigenin (**31**), luteolin (**29**), quercetin (**33**), gossypetin (**35**), cosmosiin (**36**), astragalin (**37**), linocinamarin (**41**), rutin (**42**), and nicotiflorin (**43**) (Figure 4) to facilitate analysis of their structure–activity relationship. Fourteen compounds showed HIN1-inhibitory activities, with IC_50_ values ranging from 2 to 57 µM (Table 1). Exploring the structure–activity relationship between kaempferol (**32**) and four hydroxyls, the authors further investigated the optimal position and number of hydroxyl groups on the flavonoid skeleton and showed that quercetin (**33**) activity with more hydroxyl substitutions was comparable to herbacetin activity with a 7,8-dihydroxyl group. Luteolin (**29**) with 3′ and 4′ dihydroxy groups has higher activity than kaempferol (**32**) with 3,4′-dihydroxy groups, whereas apigenin (**31**) with one less hydroxyl group is relatively less active. Gossypetin (**35**) showed 10- or 4-fold increased potency against both *Clostridium perfringens* and rvH1N1 NAs but showed similar activity against rvH1N1 NA to quercetin (**33**). Contrary to the conclusion of Du et al. [102], Lee et al. suggested [61] that the hydroxyl groups on the A or B rings are necessary for the inhibitory effect on rvH1N1 NAs. Additionally, the authors compared the activities of glycoside flavonoids and glycoside flavonoids. Astragalin (**37**) and nicotiflorin (**43**) obtained from replacing the hydroxyl group of kaempferol (**32**) showed reduced anti-fctivity. Therefore, Lee et al. [61] concluded that glycosides can cause reduced anti-influenza activity (The binding site with the target enzyme activity may be interrupted by a bulky sugar moiety).

Elderberry (*Sambucus nigra* L.) is a European native plant commonly known as black elderberry, European elderberry, European elderberry, and European black elderberry. It is rich in phenolic compounds, including phenolic acids, flavonoids, catechins, and proanthocyanidins [103]. In folk medicine, its flowers and berries are used to treat fever, cough, nasal congestion, and influenza in addition to being widely used as an anti-inflammatory, analgesic, and diuretic agent [104,105]. Zakay-Rones et al. [106] showed that elderberry extract exhibited anti-influenza activity in human clinical trials [106]. The IC_50_ of the extract for H1N1 was 252 µg/mL, while 100% inhibition of H1N1 infection was achieved at 1000 µg/mL. Roschek et al. [62] confirmed by direct binding assays that the flavonoids in elderberry extracts can bind to H1N1 influenza virus and block the ability of the virus to infect host cells upon binding. To verify the anti-influenza mode of action and in vitro anti-influenza activity of the extract, the authors synthesized 5,7,3′,4′-tetra-O-methylquercetin (**44**) and dihydromyricetin (**45**) (Figure 4). Usually, the in vitro anti-H1N1 mechanisms of polyphenols prevent intranuclear acidification [107], inhibit membrane fusion [108], inhibit the release of offspring virions [109], inhibit NA activity [109,110], and inhibit intercell replication [111]. Roschek et al. further verified that the synthetic compounds and elderberry extracts use viral envelope binding to inhibit H1N1 infection and may involve the HA domain in host cell binding and recognition. 5,7,3′,4′-tetra-O-methylquercetin (**44**) gave an IC_50_ of 0.1 µg/mL (0.4 µM) for H1N1 infection inhibition, whereas dihydromyricetin (**45**) achieved an IC_50_ of 3 µg/mL (9 µM) (Table 1) [62].

Honeysuckle (HS; *Lonicera japonica*) has been used as both medicine and food. In recent years, research has explored its various pharmacological effects, including anti-inflammatory, antibacterial, antioxidant, etc. It has significant applications and research implications in health care and disease treatment [112]. Honeysuckle has been used to treat the flu for thousands of years [113]. To clarify the main antiviral components of honeysuckle and the underlying mechanisms of its action, Li et al. [64] assessed the inhibitory activity of the total extract, organic acids extract, flavonoids extract, and acid–flavonoid mixture of honeysuckle against IAVs (H1N1 and H3N2) in vitro and in vivo. The total extract induced the lowest cytotoxicity, with a CC_50_ value of 350 µg/mL in MDCK cells. The acid–flavonoid mixture showed the most effective antiviral activity against H1N1, with an EC_50_ value of 4 µg/mL [64]. Unlike other studies, Li et al. [64] demonstrated the anti-influenza efficacy in vivo. Experiments have shown that oral administration of organic acid extracts can effectively reduce the mortality of mice infected with H1N1 virus. Oral administration of the acid extract at a dosage of 600 mg/kg/d significantly alleviated influenza virus-induced acute lung injury, improved the lung parameters, and improved the survival rate of the mice by 30%. To explore the anti-influenza mechanism of the four honeysuckle extracts, the author found through the time of drug addition (TOA) experiment that the honeysuckle extract mainly inhibited the replication and release of influenza virus into the host cells rather than viral adsorption or penetration. The influenza virus NA plays an important role by hydrolyzing sialic acid residues in progeny viruses and promoting the release of progeny virus particles. Therefore, using MUNANA, a special fluorescent substrate, they detected that four honeysuckle extracts significantly inhibited the NA activity of various influenza viruses in a dose-dependent manner. The authors found that the extract had broad-spectrum activity not only against H1N1, H3N2, H5N1, and H7N9 viral NA but also on oseltamivir-resistant mutant strains.

*Salvia plebeia* R. Br., a globally distributed edible plant, is commonly utilized in countries like India, China, Japan, and Korea as a folk remedy for various ailments, including the common cold, flu, cough, hepatitis, and hemorrhoids. [114,115,116]. To identify new antiviral lead compounds, the phytochemistry of conifer was studied. The methanolic extracts from the above-ground fraction of *S. plebeia* were extracted with CHCl3, EtOAc, and n-BuOH. The EtOAc and CHCl3 fractions were analyzed by continuous chromatography, which led to the isolation of 14 compounds. Chemical studies of the methanolic extract of *S. plebeia* isolated two new benzoylated monoterpene glycosides, polyglycosides A and B, and twelve known compounds, four flavonoids, two sesquiterpenes, four phenolics, one steroid, and one triterpene. Hispidulin (**46**), nepetin (**47**) (Figure 4), rosmarinic acid methyl ester (**48**), and luteolin (**29**) (Figure 4) exhibited moderate NA-inhibitory activity, with an IC_50_ ranging from 11 to 20 µM (seeing Table 1), compared with the inhibitory effects of the positive control (oseltamivir) (IC_50_ = 0.1 µM). Additionally, Bang et al. [63] confirmed their inhibitory effect on the virus through CPE experiments. Cell survival increased significantly at hispidulin (**46**), nepetin (**47**), and rosmarinic acid methyl ester (**48**) concentrations of 40 µM. Moreover, hispidulin (**46**) restored the chromosome condensation caused by H1N1 virus infection of MDCK cells. Among them, hispidulin (**46**), nepetin (**47**), and luteolin (**29**) had a flavonoid skeleton, hydroxyl groups on C-7 and C-4′, and α- and β-unsaturated groups on C-2, C-3, and C-4, which satisfy the structure–activity relationship of flavonoids on NA inhibition [58]. Rosmarinic acid methyl ester (**48**) is a caffeic acid ester of salvianic acid A (3,4-dihydroxyphenyllactic acid). Caffeic acid derivatives have been reported as novel influenza NA inhibitors [117].

**Figure 6:**
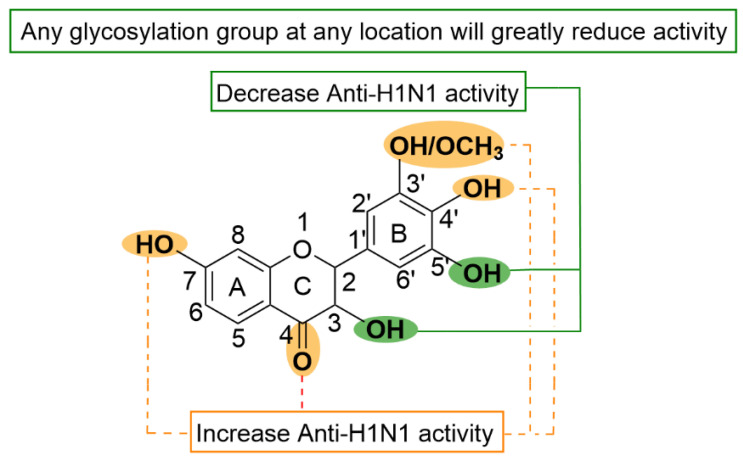
Summary of the anti-H1N1structure–activity relationships of flavonoids. Orange ellipse, groups with increased anti-H1N1 activity; orange dashed line, promoting effect; green ellipse, group with reduced anti-H1N1 activity; green solid line, inhibiting effect.

In conclusion, the above flavonoids extracted from natural products have some in vitro activities against influenza virus, and they all have anti-influenza virus activity by inhibiting NA activity (Figure 2). Therefore, the analysis of the above studies can summarize the structure–activity relationship of flavonoids on NA inhibition to elucidate the structural characteristics of flavonoids on NA inhibition, which was summarized in Figure 6. According to Liu et al. [102], in the structure–activity relationship study of flavonoid anti-influenza virus NA inhibitors, for flavonoids to have better anti-influenza virus activity, OH groups on C-7 and C-4′, a double bond between C-2 and C-3, and a carbanyl group at the C-4 position must be present. The structure of the 3′,5′-dimethylether 3-O-β-D-galactopyranoside (**18**) (Figure 4) exhibiting significant NA-inhibitory activity fully meets the above requirement. However, the IC_50_ values of demethoxymatteucinol (**19**), matteucinol (**20**), matteucin (**21**) (Figure 4), and other flavonoids with double bonds between C-2 and C-3 did not show significant changes in activity.

In exploring a new compound from the leaves of *Cleistocalyx operculatus* and its inhibitory activity against influenza A neuraminidase, Ha et al. [58] proposed that OH at the C-3′ site was replaced by methoxy, which significantly improved the inhibitory activity of flavonoids against NA. In investigating favone with six different modification groups and comparing their anti-influenza activities, Morimoto et al. [118] concluded that the modification of the B ring from the 3′ to 5′ position is important, and the hydroxyl group should preferably be at 3′ and 4′ positions rather than the 5′ position. Liu et al. [102] suggested that the increase in the number of OH groups on the B ring reduced its inhibitory effect. In 5,7,3′,4′-tetra-O-methylquercetin (**44**) and dihydromyricetin (**45**) (Figure 4), dihydromyricetin (**45**) with three hydroxyl groups on the B ring showed reduced activity against influenza virus compared with 5,7,3′,4′-tetra-O-methylquercetin (**44**). Moreover, Morimoto and Liu et al. [118,119] believed that the inhibitory effect of glycosylated flavonoid compounds would be weakened. This finding may be due to the large volume of glycosylation leading to steric hindrance or the interruption of binding sites with target enzyme activity. Therefore, we concluded that the C-7, OH, C-4′ positions, and C-4 carbon groups of flavonoid compounds are necessary to obtain good NA inhibition.

Excessive OH modification from the 3′ to 5′ sites on the B ring reduces activity. Moreover, any glycosylation group at any location will greatly reduce its activity. However, the IC_50_ values of demethoxymatteucinol (**19**), matteucinol (**20**), matteucin (**21**) (Figure 4), and other flavonoids with double bonds between C-2 and C-3 did not show significant changes in activity. In exploring the new compound of atresia leaf and its inhibitory activity against influenza A neuraminidase, Ha et al. [97] proposed that OH at the C-3′ site be replaced by methoxy, which significantly improved the inhibitory activity of flavonoids against NA. Similarly, Morimoto et al. [118], in investigating flavone with six different modification groups and comparing their anti-influenza activity, concluded that the modification of the B ring from 3′ to 5′ position is important, and the hydroxyl group should preferably be at 3′ and 4′ position rather than the 5′ position.

### 3.3. Alkaloids

Alkaloids refer to a naturally occurring class of nitrogen-containing organic compounds, excluding low-molecular-weight amines, amino acids, peptides, and proteins. They often have complex nitrogen heterocyclic structures, most of which are alkaline and can combine with acids to form salts. Alkaloids are a class of natural compounds usually with strong biological activity. They have been extensively studied for their broad-spectrum antiviral activities against different DNA and RNA viruses [119].

*Commelina communis* L. (also known as dayflower), a globally distributed herb, has been used in traditional Chinese medicine for the treatment of non-infectious fever, edema, cutin, diabetes mellitus, and other ailments [120]. Chemical components such as flavonoids, alkaloids, polysaccharides, terpenoids, and sterols have been isolated from this plant [121]. Through spectroscopic analysis, the chemical structures of the three compounds were determined as harman, homonojirimycin (HNJ) (**49**) (Figure 5), and 2,5-dihydroxymethyl-3,4dihydroxypyrrolidine. The results show that HNJ has strong antiviral activity against the influenza virus A/PR/8/34 (H1N1), with an EC_50_ value of 10 μg/mL (Table 1) and an SI value of 18. These features are comparable to those of the approved antiviral drug, ribavirin [65].

Mangroves are a unique forest ecosystem, mainly distributed in tropical and subtropical intertidal zones, with rich biodiversity and abundant actinomycetes [70,122]. To search for bioactive products from mangrove actinomycetes, a new endophytic actinomycete was isolated from the roots of *Xylocarpus granatum* (Meliaceae) and identified as *Jishengella endophytica* 161,111 [123]. Wang et al. [66] chemically studied the EtOAc extract of strain 161,111 and identified 13 compounds. These 13 compounds were tested for their antivirus effects on H1N1 using the CPE inhibition assay, and ribavirin was used as the positive control, with an IC_50_ value of 23 µg/mL. The results showed that perlolyrine (**50**), 1-hydroxy-β-carboline (**51**), lumichrome (**52**), and 1H-indole-3-carboxaldehyde (**53**) (Figure 5) have moderate anti-H1N1 activity with semi-inhibitory concentrations of 38, 25, 40, and 46 μg/mL, respectively (seeing Table 1). The cytotoxicity assay revealed that perlolyrine (**50**), 1-hydroxy-β-carboline (**51**), and 1H-indole-3-carboxaldehyde (**53**) exhibited mild toxicity towards MDCK normal cells, with CC_50_ values of 116.3 ± 12.1, 403.2 ± 31.4, and 522.5 ± 24.5 μg/mL, respectively.

Since the 1980s, studies have been conducted on marine sponges belonging to the class Calcarea, producing large amounts of bioactive alkaloids containing imidazole heterocyclary substitutions [67]. This alkaloid has been reported to have cytotoxic [124,125,126], antimicrobial [127], and antifungal [128] properties. Additionally, they possess leukotriene B4 receptor [129] and epidermal growth factor receptor [130] antagonist activities. According to a chemical study by Gong et al. [67] on sponge-heterologous organisms collected from the South China Sea, a new imidazole alkaloid named naamidine J—along with four known ones: naamidine H, pyronaamidine, leucettaamine B, and leucettamine C (**54**)—was identified (Figure 5) [131,132]. Of these five alkaloids, leucettamine C (**54**) exhibited weak anti-H1N1 activity with an inhibition ratio of 33% (Table 1). The cytotoxicity assay revealed that leucettamine C (**54**) did not exhibit cytotoxicity on human leukemia (K562), acute myeloid leukemia (HL-60), cervical cancer (HeLa), and lung adenocarcinoma (A549) cells.

*Peganum harmala* L. (family *Zygophyllaceae*) is a perennial, hairless plant widely grown in China, the Middle East, India, and South America [133,134,135]. Moradi et al. [68] found that the crude extract inhibited the replication of type A PR8 virus in this cell line, with an IC_50_ value of about 10 (95% CI: 7–11) µg/mL (Table 1). The total alkaloids of this extract had antiviral activity, with an IC_50_ value of about 6 (95% CI: 4–9) µg/mL (Table 1), which was better than that of the crude extract. To explore the mode of action of the extract against influenza virus replication, Moradi et al. [68] studied the mode of action of viricidal activity against influenza virus replication using tests, including a coagulation inhibition test, time of addition test, RNA replication, Western blot analysis, and RNA polymerase blocking assay. The results suggested that harmala seed extracts may reduce NP levels and viral polymerase activity, thereby affecting the RNP complex activity and subsequently inhibiting viral RNA transcription and replication.

Papaverine (**55**) (Figure 5) is a non-narcotic opiate alkaloid. Medicinal papaverine is used as a smooth muscle relaxant for the treatment of vasospasm and erectile dysfunction, and its mechanism of action is the inhibition of phosphodiesterase 10A [136,137,138]. Papaverine has antiviral activity, with an IC_50_ value of about 17 µM (Table 1). Papaverine exhibited a dose-dependent inhibition of influenza virus strains used in this study (A/WSN/33, A/Udorn/72, A/Eq/2/Miami/1/63, B/Lee/40, and B/MD/59) [69]. Aggarwal et al. [69] identified the inhibition step in the viral life cycle through the time of addition (TOA) and time of elimination (TOE) experiments. The results collectively suggest that the inhibitory effect of papaverine occurs late in the influenza virus infection cycle.

Aggarwal et al. [69] evaluated the effects of papaverine on the activities of HA and NA proteins and found that papaverine had no effect on the activities of HA or NA surface glycoproteins, proving that papaverine did not interfere with the entry or release steps of the virus life cycle. Viral RNA synthesis was analyzed by semiquantitative reverse transcriptase PCR (RT-PCR). The viral RNA grew in the presence of a specified concentration of papaverine, and the results showed that papaverine did not affect the synthesis of influenza viral RNA. HEK293T cells were infected with A/WSN/33 virus and treated with papaverine or DMSO. Western blotting analysis showed that the presence of papaverine reduced the phosphorylation of MEK and ERK (Figure 2). However, the amount of MEK and ERK remained the same. Therefore, the results suggest that papaverine alters the activation of MEK/ERK pathway in 293T cells. Papaverine was found to affect the morphology of influenza virus by inhibiting nuclear export of the viral genome (Figure 2).

*Isatis indigotica* Fort. (Cruciferae) is a widely grown medicinal plant. The dried leaves and roots of the plant, known in China as “da qin ye” and “ban lan gen”, are used in traditional Chinese medicine to treat influenza and other infections [139]. Water extracts from *I. indigotica* leaves and roots were studied. Fifty-seven new alkaloids were isolated from *Radix isatidis*, of which 22 alkaloids were indoles and bisindole alkalosides. Seven indole alkaloid glycosides containing the 1′-(4′-hydroxy-3′,5-dimethoxyphenyl) ethyl unit were isolated from the *I. indigotica* leaf (da qing ye) decoction by Guo et al. [140]. Isatidifoliumosides (**56**)/epiisatidifoliumosides C (**57**) (Figure 5) in the 3:2 ratio exhibited antiviral activity against influenza virus H1N1 PR8, with an IC_50_ of 65 µmol/L (seeing Table 1) (the positive control ribavirin, IC_50_: 54 µmol/L).

### 3.4. Terpenoid Derivatives

Terpenoids are compounds derived from meglutaric acid and their derivatives whose molecular framework is based on isoprene unit (C5 unit). Terpenoids have anti-tumor, anti-inflammatory, antibacterial, and antiviral properties. They prevent cardiovascular and cerebrovascular diseases and are one of the most abundant compounds in natural products [141].

**Figure 7:**
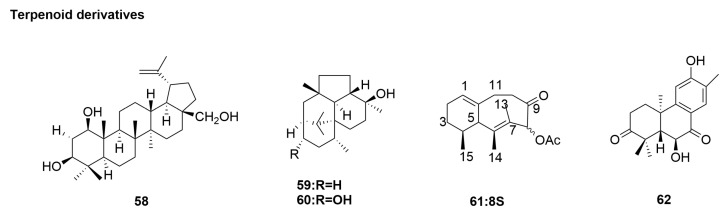
Terpenoid derivatives with activity against H1N1.

The genus *Sonneratia*, consisting of nine mangrove plant species in the family *Sonneratiaceae*, is widely distributed in tropical and subtropical regions. These plants exhibit diverse biological activities and have been traditionally employed for treating ailments including asthma, ulcers, hepatitis, hemorrhoids, sprains, and bleeding [142]. Chemical studies of the aerial parts of the mangrove plant, *Sonneratia paracaseolaris*, yielded five new triterpene paraproteins: proteins A–E and 12 known analogues. Additionally, the CPE test was used to evaluate the antiviral activity of all isolates against the influenza A H1N1 virus (IAV) [142]. Only paracaseolins A (**58**) (Figure 7) showed significant anti-H1N1 viral activity, with an IC_50_ value of 28 µg/mL, which was close to that of the ribavirin positive control, with an IC_50_ value of 25 µg/mL (Table 1). The other compounds showed no activity with inhibition rates of <50% at 50 µg/mL [71].

*Ganoderma lingzhi* (formerly called *Ganoderma lucidum* [143,144]), an oriental fungus, has been used in traditional Chinese medicine for thousands of years [145]. The lanostane-type triterpenoids are the main bioactive components of *G. lingzhi*, and they have been reported to have various physiological activities, including anticancer, immunomodulatory, antihypertensive, antiandrogenic, antidiabetic, and antiviral properties [146,147]. The direct effect of the hot water extract of *G. lingzhi* on NA was evaluated by Zhu et al. using an in vitro NA-inhibition assay with four different influenza A virus subtypes [72]. The extract strongly inhibited the activities of NAs derived from the influenza A virus subtype H1N1, with an IC_50_ value of 15 µg/mL. Moreover, Zhu et al. [72] studied the anti-influenza effects of *G. lucidum* hot water extract in two ways, i.e., intranasally and orally, in a convalescent mouse model. Their results showed that intranasal injection of *G. lingzhi* hot water extract had a direct inhibitory effect on influenza A virus and could effectively alleviate weight loss during influenza infection in mice. In further NA-inhibition experiments, the extracts showed strong inhibitory effects on NA activity of influenza A virus H1N1 subtype (IC_50_ = 15 µg/mL) and H5N1 subtype (IC_50_ = 2 µg/mL) (Table 1). However, no inhibitory effect was observed on NA activity of the other two influenza A viruses, H3N2 and H7N9 subtypes (IC_50_ >900 µg/mL).

In the process of screening for novel anti-influenza virus agents from microbial metabolites, two novel diterpene compounds, wickerols A (**59**) and B (**60**) (Figure 7), were isolated from the culture broth of a fungus, *Trichoderma atroviride* FKI-3849, by Yamamoto et al. [73]. Wickerol showed a strong antiviral activity against the A/H1N1 flu virus (A/PR/8/34 and A/WSN/33 strains), with an IC_50_ value of 0.1 mg/mL (Table 1), but it was not active against the A/H3N2 virus [73].

Located in the tropics and subtropics, the South China Sea has a vast sea area and rich marine biological resources. Since the 1980s, many compounds with novel structures, including alkaloids and terpenoids, have been isolated from various corals collected in the South China Sea, and the isolated chemical components have been studied [57,148,149,150,151,152]. *Lemnalia sp.* (No. xssc2011907) is a soft coral collected from the coast of the Xisha Islands in the South China Sea. A chemical study of acetone extract by Yan et al. [74] revealed thirteen structurally different terpenoids, six new diterpenoids, and three known related compounds. Four sesquiterpenes showed anti-H1N1 viral activity at 30 µM with 77.6–100% inhibition, whereas the novel sesquiterpene lineolemnenes F (**61**) (Figure 7) showed activity with an IC_50_ of 6 µM (Table 1).

*Celastrus aculeatus* Merr. is an evergreen vine widely distributed in southern China [151]. In folk culture, the roots and stems of this plant are often used in traditional Chinese medicine to treat diseases such as rheumatoid arthritis, gout, cholecystitis, nephritis, and hypertension, and they have many biological activities [152]. Previous studies have demonstrated the inhibitory effect of triterpenes and diterpenes (including phenolic and indexing diterpenoids) on H1N1 virus A [153]. The phytochemical exploration of *C. aculeatus* was performed to identify new antiviral lead compounds by Chen et al. [75]. Antiviral activities of the compounds were evaluated. The EtOAc-soluble fraction of the methanolic extract of the whole plants of *C. aculeatus* was subjected to silica gel chromatography and then purified by semipreparative HPLC to yield seven diterpenoids. Antiviral activities of the compounds were preliminary evaluated on the A/PR/8/34 (H1N1) strain using oseltamivir as a positive control. The antiviral activity assay showed that aculeatusane A (**62**) (Figure 7) is active against A/GZ/GIRD07/09 (H1N1), with an IC_50_ of 23 µM and SI of 7 µM (Table 1).

### 3.5. Phenol Derivatives

Phenolic compounds are compounds formed by the combination of one or more aromatic rings with one or more hydroxyl groups. Phenolic compounds are secondary metabolites of plants, which have been confirmed to have various significant biological activities [154], including anti-cancer, analgesic, anti-inflammatory, and antibacterial effects. Additionally, they have biological activities against diabetes mellitus, cardiovascular and cerebrovascular diseases, and influenza A virus.

**Figure 8:**
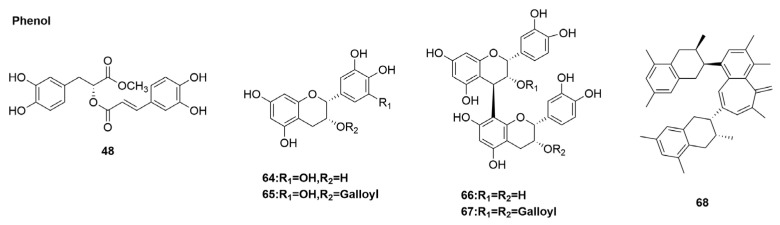
Phenol compounds with activity against H1N1.

*Calotropis gigantea* (Asclepiadaceae) is a shrub found in East and Southeast Asia, and its bark and leaves have been traditionally used in Chinese folk medicine [154]. A series of bioactive secondary metabolites, such as cardiosteroids, triterpene alcohols, alkaloids, and flavonoids, have been isolated from different parts and have shown pharmacological activities such as analgesic, sedative, anti-inflammatory, and antidiarrheal effects [155,156,157,158]. A new lignan glycoside, (+)-pinoresinol 4-O-[6″-O-vanilloyl]-β-D-glucopyranoside (**63**) (Figure 8), was isolated from the latex of *Calotropis gigantea* (Asclepiadaceae) by Parhira et al. [76]. Three isolates and one authentic compound were screened for A/PR/8/34 (H1N1)-inhibitory activity by CPE inhibition assay on MDCK cells. (+)-pinoresinol 4-O-[6″-O-vanilloyl]-β-D-glucopyranoside (**63**) showed an inhibitory activity against A/PR/8/34 (H1N1). An antiviral activity assay showed that (+)-pinoresinol 4-O-[6″-O-vanilloyl]-β-D-glucopyranoside is active against A/GZ/GIRD07/09 (H1N1), with an IC_50_ of 25 µM (Table 1). Parhira et al. [158] tested the antiviral mechanism of (+)-pinoresinol 4-O-[6″-O-vanilloyl]-β-D-glucopyranoside through TOA. The results showed that, unlike oseltamivir, it blocked the release of progeny virions and blocked the later stage of viral replication. The antiviral mechanism of (+)-pinoresinol 4-O-[6″-O-vanilloyl]-β-D-glucopyranoside is by inhibiting the early stages of influenza virus replication. Furthermore, it inhibited virus-induced NF-kB activation in a dose-dependent manner [159]. These results suggest that (+)-pinoresinol 4-O-[6″-O-vanilloyl]-β-D-glucopyranoside prevents influenza virus replication by inhibiting NF-kB activation, leading to impaired nuclear RNP output.

Tea polyphenols are a general group of natural polyhydroxyphenolic compounds extracted from tea, which have a variety of physiological functions such as antioxidant, anti-cancer, anti-radiation, anti-aging, cardiovascular disease prevention, and antiviral activity [160]. The in vitro antiviral activity of 13 tea polyphenols against influenza A and B viruses was comprehensively investigated. The results showed that five compounds, namely (−)-epigallocatechin (EGC) (**64**), (−)-epigallocatechingallate (EGCG) (**65**), procyanidin B-2 (**66**), procyanidin B-2 3,3′-di-O-gallate (**67**), and theaflavin (**68**) (Figure 8), had anti-influenza A virus activity [77]. Based on these results, Yang et al. [77] found that dimeric flavonoid 3-ols without gallic groups, such as theaflavin (**68**) and procyanidin B-2 (**66**), had a broader spectrum of anti-influenza virus activity (Table 1). The dimer molecule showed stronger activity against influenza virus than catechin monomer. Additionally, compared with methylated EGC, the EGC phenol hydroxyl group (**64**) on the B ring played an important role in anti-influenza A virus activity. The antiviral effects of tea catechin and its dimer on influenza A and B viruses may have the following two mechanisms: (1) blockage of the binding of virus to cell receptors in the early stage of viral infection and (2) inhibition of viral replication after the entry of virus [77]. Theaflavin (**68**) and procyanidin B-2 (**66**), two dimers of flavan-3-ols, both inhibit influenza A and B viruses, possibly due to the combination of the two mechanisms mentioned above.

### 3.6. Polysaccharides

Polysaccharides are carbohydrate substances with complex and large molecular structures, formed by condensation of several monosaccharide molecules and loss of water. Polysaccharides are natural macromolecular substances composed of ten or more monosaccharides bonded by glycosidic bonds, which are the material basis of active ingredients in traditional Chinese medicine. Natural polysaccharides are widely found in nature and have various pharmacological effects, such as anti-tumor and immune regulatory effects. The following discussion focuses on some polysaccharides isolated from natural products with inhibitory activity against influenza A viruses.

Naturally occurring sulfated exopolysaccharides have exhibited varying degrees of inhibitory activity, probably depending on their molecular weight and degree of sulfation [161,162,163]. p-KG03, is found in the marine dinoflagellate *Gyrodinium impudium* [164], and the results from virus-infected cells showed that influenza A viruses were sensitive to p-KG03, with EC_50_ values of 0.5 and 0.2 µg/mL against PR8 and TW, respectively (Table 1). Kim et al. [78] demonstrated that p-KG03 has anti-H1N1 influenza virus activity in MDCK cells, with an EC_50_ of 0.2–0.5 µg/mL (IS > 200). In the mechanism study, the inhibition of influenza virus replication was greatest within 0–6 h after infection, indicating that the compound mainly targets the adsorption and internalization steps in the early stages of the viral replication cycle (Figure 2). The binding experiments and fluorescence microscopy showed that the antiviral activity of p-KG03 was directly related to the interaction between p-KG03 and viral particles.

Jiao et al. [79] extracted polysaccharides from two types of brown algae (*A. nodosum* and *F. Vesiculosus*) using a sequential extraction method. Methanol was used for the initial extraction to remove non-polysaccharide components soluble in methanol. Subsequently, cold water, hot water, and alkaline solution were used for separate extractions. The antiviral activity of polysaccharides isolated from various Atlantic seaweed species against influenza was then evaluated. The results demonstrated that all extract of polysaccharides from *A. nodosum* and *F. vesiculosus* exhibited significant inhibitory effects on the A/PR/8/34 (H1N1) influenza virus and displayed high or medium antiviral activities against the influenza A/PR/8/34 virus (Table 1). In another study, Yu et al. [80] extracted polysaccharides in three conditions, namely 85% ethanol at 80℃, 20 volumes of distilled water at 80℃, and 10 volumes of 4% NaOH at 60℃, from the red algae *Eucheuma denticulatum* and evaluated the anti-influenza A (H1N1) viral activity of these three extracts using the Madin–Darby canine kidney cell model. Among them, ethanol extract had a good activity against H1N1 virus, with a semi-inhibitory concentration of 277 μg/mL and 52% inhibition against H1N1 virus at 250 μg/mL (Table 1). The semi-inhibitory concentration of ι -carrageenan water extract was 366 μg/mL, while NaOH extract had less anti-H1N1 virus activity (IC_50_ > 430 μg/mL) (Table 1). The available data suggest that hybrid carrageenan ethanol extract obtained in the future could be used as a potential anti-H1N1 viral inhibitor. The difference in the effectiveness of ethanol extract and water extract against the H1N1 virus may be due to the different levels of acidification and viscosity.

The antiviral activity of polysaccharides has been reported to be related to monosaccharide composition, molecular weight, and sulfation level [165,166,167,168]. Ray et al. [165] found that the degree of sulfation (DS) of the polymer was an important parameter for the antiviral activity of polysaccharides, and the higher the DS, the better the antiviral effect [169]. From a structural point of view, highly charged molecules effectively interfere with electrostatic interactions between the positively charged regions of viral glycoproteins and the negatively charged heparan sulfate (HS) chains of glycoprotein receptors on the cell surface. Sulfated polysaccharides with sulfate content higher than 20 (mol%) showed obvious antiviral activity. A higher average molecular weight (MW) indicated a higher antiviral activity. High-molecular-weight polysaccharides can significantly inhibit the binding and entry of viral receptors, but low-molecular-weight polysaccharides can better reduce the intercellular transmission of viruses [165].

### 3.7. Miscellaneous Compounds

**Figure 9:**
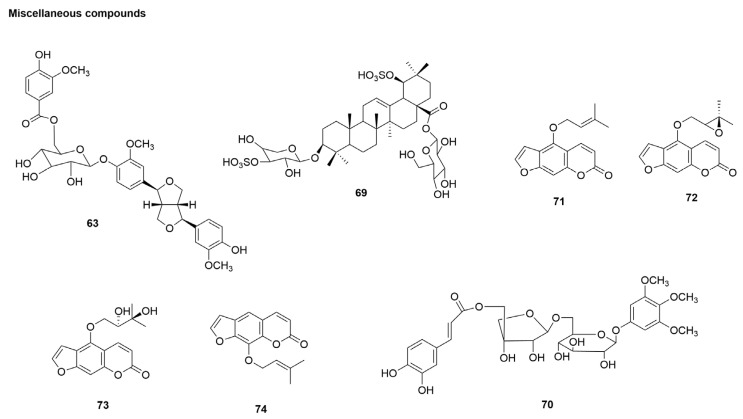
Phenol and miscellaneous compounds with activity against H1N1.

In addition to the natural compounds mentioned above, a number of coumarins, steroidal saponins, and other compounds have been found to have anti-influenza activities. The roots of *Ilex asprella* (Hook. et Arn.) Champ. ex Benth. (Aquifoliaceae) are widely used in Chinese medicine to treat diseases such as influenza, amygdalitis, and pertussis [81]. A novel saponin containing sulphonic groups, namely asprellcoside A (**69**) and 3,4,5-trimethoxyphe β-D-5-O-caffeoyl-apiofuranosyl-(16) -β-D-Glucopyranoside (**70**) (Figure 9), was isolated from the roots of *Ilex asprella* and inhibited influenza virus strain A/PuertoRico/8/1934 (H1N1) strongly, with EC_50_ values of 4.1 and 1.7 mM (Table 1), respectively. Moreover, both compounds inhibited the secretion of IP-10, with EC_50_ values of 7 and 3 mM, respectively. In ref. [81], asprellcoside A (**69**) and 3,4,5-trimethoxyphe β-D-5-O-caffeoyl-apiofuranosyl-(16) -β-D-Glucopyranoside (**70**) showed strong anti-inflammatory effects on influenza-related tests and reduced levels of the chemokine IP-10 in H1N1-infected cells (Figure 9). However, further studies are needed on their antiviral mechanisms, effects on the virus life cycle, and targets. Toxicity assays showed that asprellcoside A (**69**) and 3,4,5-trimethoxyphe β-D-5-O-caffeoyl-apiofuranosyl-(16) -β-D-Glucopyranoside (**70**) showed no cytotoxicity (CC_50_ > 100 μM).

Lee et al. [82] identified four active furanocoumarins from 70% ethanol extract of *Angelica dahurica* (*A. dahurica*) root through the bioactivity-guided isolation: isoimperatorin (**71**), oxypeucedanin (**72**), oxypeucedanin hydrate (**73**), and imperatorin (**74**) (Figure 9). Among them, oxypeucedanin showed a significant CPE-inhibitory effect, which was stronger than that of the positive control, ribavirin, against H1N1, with an EC_50_ of 6 μM (Table 1). Oxypeucedanin inhibited the synthesis of NA and NP in a dose-dependent manner. Lee et al. [82] further studied the mechanism of oxypeucedanin and found that it interfered with the synthesis of NP and NA at the early stage of viral replication; it exerted anti-influenza activity but did not affect the virus’ entry into host cells, emergence, and release. The molecular docking analysis predicted the role of oxypeucedanin in polymerase acidic protein, which inhibits viral mRNA transcription and thus viral protein synthesis. Additionally, oxypeucedanin reduced H1N1-induced apoptosis by inhibiting the Bax/casepase-3 pathway (Figure 2).

### 3.8. Derivatives of Natural Products

**Figure 10:**
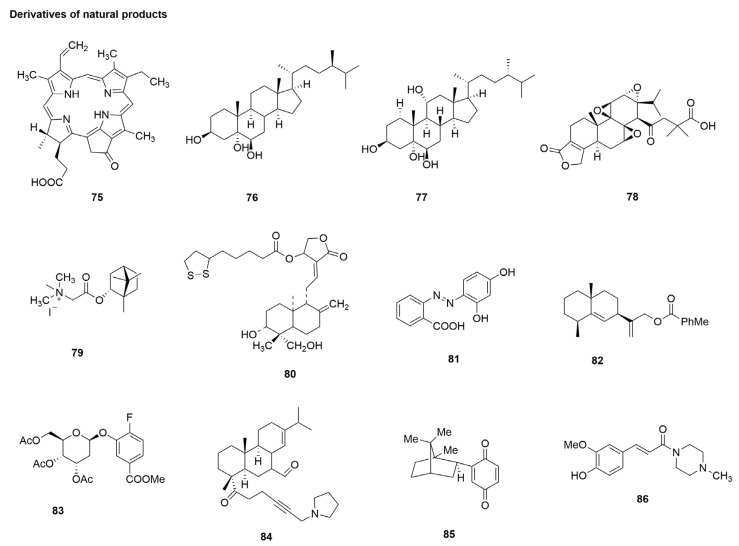
Derivatives of natural products with activity against H1N1.

Natural products have long been an important source of many marketed drugs, with studies showing that 10% of drugs on the market are unmodified natural products, and 29% are derivatives (hemicompounds) [170]. The natural product gentiopicroside (GPS) is an annular ether terpenoid, which is widely found in gentian (*Gentiana manshurica* Kitag.), which is one of its active components. Although it has been shown to have anti-inflammatory activity [171], it is still very far from clinical use. To improve its bioavailability and lipid solubility, Wu et al. [83] designed and synthesized a series of new gentiside derivatives, followed by in vitro inhibition of influenza virus biological evaluation of all synthesized compounds. 2′,3′,6′-Tri-O-benzoyl-4′- O-methylsulfonyl gentiopicroside (**75**), 4′-fluoro-4′-deoxy gentiopicroside (**76**), and 2′,3′,6′-Tri-O-benzoyl-4′,5′-olefin gentiopicroside (**77**) (Figure 10) showed significant activity against influenza viruses, with IC_50_ values of 39.5 μM, 45.2 μM, and 44.0 μM, respectively (Table 1) [83]. The authors further evaluated their cytotoxicity in MDCK cells, with results indicating no cytotoxic effects on uninfected MDCK cells at a concentration of 50µM. Triptolide (TP) is one of the main active substances of mine, a commonly used Chinese medicine in China. Although recent studies have demonstrated that TP has antiviral effects [172], TP has poor water solubility and rapid elimination in vivo, and high concentrations of TP can cause multiple organ toxicity, which hinders the development of its clinical application. The TP derivatives were designed and synthesized, and their activity against influenza A viruses was evaluated by Jiang et al. [84]; the results show that 4-(((5bS, 6aS, 7aR, 8R, 8aS, 9aS, 9bS, 10aS, 10bS)-8a-isopropyl-10 bmethyl-3-oxo 1, 2,3, 5,5b, 6,6a, 8,8a, 9a, 9b, 10b dodecahydrotris(oxireno) [2′, 3′:4b, 5;2″, 3″:6, 7;2, 3‴:8a, 9] phenanthrol [1, 2-c] furan-8-yl)oxy)-2, 2-dimethyl-4 oxobutanoic acid (TPDMSA) (**78**) (Figure 10) is an anti-influenza virus agent mainly by inhibiting the nuclear export of the influenza virus vRNP, acting at the late stage of the H1N1 viral replication cycle, with significant inhibition of NP activity late in the H1N1 viral replication cycle by binding to the NP tail loop active site. The EC_50_ value of TPDMSA (**78**) for A/WSN/33 (H1N1) was 3.24 μM (Table 1). Anastasiya S. Sokolova et al. found high anti-influenza activity of camphor imine derivatives in a previous study [173,174,175]. A series of compounds containing a 1,7,7-trimethylbicyclo [2.2.1] heptane fragment was then studied. The results showed that N, N, N-Trimethyl-2-oxo-2-((1S,2R,4S)-1,7,7-trimethylbicy-clo [2.2.1] heptan-2-yloxy) ethanaminium iodide (**79**) (Figure 10) exhibited potent anti-influenza activity against influenza A (H1N1) virus in vitro, with an IC_50_ = 2.4µM, and showed low toxicity (CC_50_ = 1311µM) (Table 1) [85]. Chen et al. [86] synthesized a series of andrographolide derivatives and investigated their anti-influenza activity, which showed that AL-1 (14-alpha-lipoyl andrographolide) (**80**) (Figure 10) showed potent anti-influenza activity against H1N1 virus in vitro, with EC_50_ = 7.2 µM. Li et al.’s [87] evaluation of the anti-influenza virus activity of the 50 synthesized resveratrol derivatives showed that R42 (**81**) (Figure 10) not only had high inhibitory activity against NA, with an IC_50_ = 3.56 µM (Table 1), but also against influenza virus in MDCK cells, implying that the mechanism of anti-influenza virus activity may be through NA inhibition. Lin et al. [88] designed 23 pterodontic acid derivatives for the structural modification of pterodontic acid derivatives isolated from *Laggera pterodonta* (DC.) Benth. The results showed that compound 15 (**82**) (Figure 10) had a better activity against H1N1 virus (IC_50_ = 9.92 µM). Xue et al. [89] synthesized a series of substituted arylglycosides as analogues of gastrodin and evaluated their anti-influenza activity. The most effective compound was methyl 4-fluoro-3-((2S,3R,4S,5R,6R)3,4,5-triacetoxy-6-(acetoxymethyl)-tetrahydro-2H-pyran-2-yloxy) benzoate (**83**)] (Figure 10), which showed significant inhibitory activity against influenza A virus A/FM/1/47(H1N1), with an IC_50_ = 34.4 μM. Tret‘yakova et al. [90] synthesized a series of compounds of the terpene series by introducing heterocyclic fragments into the diterterpene skeleton. Among them, compound 12 (**84**) (Figure 10), containing pyrrolidine fragments, showed the least toxicity to influenza A virus. The antiviral activity was the highest (IC_50_ = 5.0μM, CC_50_ = 643μM) (Table 1). Chukicheva et al. [91] performed a preliminary evaluation of the antiviral activity of terpenophenols and their N or O-containing derivatives, in which 2-(1,7,7-Trimethylbicyclo [2.2.1]hept-exo-2-yl)cyclohexa-2,5-dien-1,4-dione (**85**) (Figure 10) was the most active influenza NA inhibitor, with an IC_50_ = 0.5 µM (Table 1). Cui et al. [92] designed and synthesized four series of ferulic acid derivatives, among which the most effective compound was E)-3-(4-Hydroxy-3-methoxyphenyl)-1-(4-methylpiperazin-1-yl)- prop-2-en-1-one (**86**) (Figure 10), with an IC_50_ = 12.77 ± 0.47 μg/mL (Table 1).

## 4. Druggability

Natural products have long been recognized as important sources of clinically innovative drugs and valuable sources of drug design not only in the anti-influenza field but also in areas such as anti-tumor research [176]. Although natural products contain large amounts of biologically active groups, there are many difficulties in developing them into clinical drugs [177]. Compared with synthetic drugs, natural products usually have medicinal disadvantages such as poor water solubility, poor bioavailability, and weak biological relative activity. Therefore, through the traditional Chinese medicine systems pharmacology database and analysis platform (TCMSP) website, we summarized the ADME-related properties of some natural compounds to visually assess their medicinal properties (Table 2).

Lipinski’s five rules posit that molecular weight (MW < 500), lipophilicity (Alog*P* < 5), hydrogen bond donor count (Hdon < 5), hydrogen bond acceptor count (Hacc < 10), and the number of rotatable bonds (RBN < 10) are essential criteria for assessing drug likeness [178,179]. Excessive molecular weight can impact drug absorption in the intestine. With the exception of kaempferol-3-sophoroside, quercetin-3-sophoroside, rutin, nicotiflorin, and procyanidin B-2, the molecular weights (MW) of other compounds are all below 500 Daltons (Da). Compounds in Table 2 exhibit favorable lipophilicity, with the Alog*P* of all < 5. Perlolyrine, 1H-indole-3-carboxaldehyde, hispidulin, and others meet Lipinski’s criteria by having favorable hydrogen bond donor count (Hdon < 5) and hydrogen bond acceptor count (Hacc < 10) as well as and RBN < 10. OB is one of the most critical pharmacokinetic parameters, serving as a key indicator of the effectiveness of drug entry into the human circulatory system. Adequate oral bioavailability is fundamental for compounds to exhibit pharmacological activity. Luteolin (36.16%), perlolyrine (65.95%), papaverine (64.04%), and others in Table 2 demonstrate relatively high oral bioavailability. Moreover, except for 1H-indole-3-carboxaldehyde, DL (drug-likeness) is above 0.18, indicating good drug-likeness.

However, this is only a small part of the natural products against H1N1, and more of these demonstrate less druggability. Nonetheless, natural products have received much attention due to their abundant sources and potential anti-H1N1 applications. Therefore, researchers still need to overcome the problems of low solubility, poor stability, and low bioavailability. Nanomedicine applications can well solve these problems. Nanoparticle technology helps to improve the solubility, stability, bioavailability, target specificity, and bioactivity of natural products through different encapsulation techniques [180,181].

## 5. Conclusions and Outlooks

The emergence of influenza A virus has caused severe harm to individuals, societies, and countries. Currently, there are two strategies to combat influenza viruses: vaccine and antiviral drugs. To date, WHO believes that influenza virus vaccination remains the best way to control the spread of influenza in humans [182]. However, use of the vaccine has been limited by side effects and difficulties in transportation and storage [183]. Therefore, drug therapy is the most effective means to control the spread of influenza virus. Because influenza A virus is prone to antigenic mutations, inhibitors targeting virus surface proteins (such as NA inhibitors and M2 ion channel inhibitors) are prone to developing resistance despite the emergence of new antiviral drugs. The active ingredients in natural products and natural medicine have always been a hotspot of new drug development. They can play a role in preventing and treating influenza A by improving immune function in a number of ways. Therefore, natural products have unique advantages and broad prospects in the fight against influenza A.

In this review, different types of natural anti-influenza A products were classified. They included marine fungal metabolites, flavonoids, alkaloids, terpenoids, phenols, and polysaccharides, and their anti-influenza A activities were discussed. The inhibitory effect of flavonoids on viral NA causes flavonoid compounds to exhibit strong anti-influenza A activity, and the structure–activity relationship was reviewed clearly. Alkaloids have been reported as inhibitors of various viral protein synthesis, primarily targeting protein synthesis during the virus’ life cycle. In studies on the anti-influenza activity of papaverine, it was found to inhibit viral infection later in the life cycle of influenza virus. However, the reason underlying the changes in morphology of influenza virus is still not exactly understood. Interestingly, to find highly effective antiviral active ingredients from natural products, several marine natural products have gained attention, providing new ideas for the development of novel anti-influenza A virus drugs.

In conclusion, natural products are an important source for the discovery of lead compounds and drug candidates against influenza A virus. However, the current studies on the anti-influenza A (H1N1) virus activity of natural products mainly focus on the screening of active ingredients and in vitro studies. Therefore, we believe that the mechanism of anti-influenza A (H1N1) requires more in-depth study, and more in vivo and clinical studies are needed to demonstrate anti-influenza A activity to further study effective and safe anti-influenza A (H1N1) drugs. Moreover, many natural products still need to overcome the problems of low solubility, poor stability, low bioavailability, and other drug-formation properties. Although natural products combined with nanoparticle technology can solve these problems, they are mostly in the preclinical research stage and require further clinical research.

## Figures and Tables

**Table 1:** Anti- H1N1 phytochemicals from natural resources.

No	Natural Resource	Active Compound		Strain	Activities	Ref.
**1**	*Paratetilla* sp. sponge-derived fungus, *Penicillium sclerotiorum* OUCMDZ-3839	Sclerotiorin E		H1N1 A/PR/8/34	IC_50_ = 79 μM	[51]
**2**		(+) Sclerotiorin		H1N1 A/PR/8/34	IC_50_ = 129 μM	
**3**		TL-1-monoactate		H1N1 A/PR/8/34	IC_50_ = 115 μM	
**4**		Ochrephilone		H1N1 A/PR/8/34	IC_50_ = 151 μM	
**5**		8-acetyldechloroisochromophilone III		H1N1 A/PR/8/34	IC_50_ = 91 μM	
**6**		Scleratioramine		H1N1 A/PR/8/34	IC_50_ = 134 μM	
**7**		Isochromophilone IX		H1N1	IC_50_ = 157 μM	
**8**	Marine-derived fungus *Aspergillus terreus* SCSGAF0162	Asperterrestide A		A/WSN/33 (H1N1)	IC_50_ = 15 μM	[52]
**9**	Marine-derived fungus *Aspergillus terreus* OUCMDZ-1925	Rubrolides S		H1N1 A/PR/8/34	IC_50_ = 87 µM	[53]
**10**	*Aspergillus* sp. SCSIO XWS02F40	Asteltoxin E		H1N1	IC_50_ = 4 µM	[54]
**11**	*Streptomyces* sp. OUCMDZ-3434	Wailupemycin J		H1N1	47.8% inhibition at 50 μg/mL	[55]
**12**		R-wailupemycin K		H1N1	42.5% inhibition at 50 μg/mL	
**13**		5-deoxyenterocin		H1N1	60.6% inhibition at 50 μg/mL	
**14**	M. *senhousei*	Pyropheophoride a		H1N1 A/PR/8/34	IC_50_ = 0.17 µg/mL	[56]
**15**	The South China Sea soft coral *Sarcophyton* sp.	(24R)-methylcholest-7-en-3β,5α,6β-triol		H1N1 A/PR/8/34	IC_50_ = 19.6 µg/mL	[57]
**16**	The South China Sea soft coral *Sarcophyton* sp.	(24S)-ergost-3β,5α,6β, 11α-tetraol		H1N1 A/PR/8/34	IC_50_ = 36.7 µg/mL	[57]
**17**	*Cleistocalyx operculatus* leaves	2′,4′dihydroxy-6′-methoxy-3′,5′-dimethylchalcone		H1N1 A/PR/8/34	IC_50_ = 8 μM	[58]
**18**		Myricetin-3′,5′-dimethylether 3-O-β-D-galactopyranoside		H1N1 A/PR/8/34	IC_50_ = 9 μM	
**19**	*Pentarhizidium orientale*	Demethoxymatteucinol		H1N1 A/PR/8/34	IC_50_ = 30 µM	[59]
**20**		Matteucinol		H1N1 A/PR/8/34	IC_50_ = 25 µM	
**21**		Matteucin		H1N1 A/PR/8/34	IC_50_ = 24 µM	
**22**		Methoxymatteucin		H1N1 A/PR/8/34	IC_50_ = 25 M	
**23**		3′-hydroxy-5′-methoxy-6,8dimethylhuazhongilexone		H1N1 A/PR/8/34	IC_50_ = 24 µM	
**24**	Bee pollen	Kaempferol-3-sophoroside		H1N1	IC_50_ = 86 µM	[60]
**25**		Kaempferol-3-neohesperidoside		H1N1	IC_50_ = 56 µM	
**26**		Kaempferol-3-sambubioside		H1N1	IC_50_ = 45 µM	
**27**		Kaempferol-3-glucoside		H1N1	IC_50_ = 36 µM	
**28**		Quercetin-3-sophoroside		H1N1	IC_50_ = 88 µM	
**29**	Bee pollen/*Rhodiola rosea*/*Salvia plebeia* R. Br.	Luteolin		H1N1	IC_50_ = 11-18 µM	
**30**	Bee pollen	Chelianthifoline		H1N1	IC_50_ = 101 µM	
**31**	*Rhodiola rosea*	Apigenin		rvH1N1	IC_50_ = 33 µM	[61]
**32**		Kaempferol		rvH1N1	IC_50_ = 11 µM	
**33**		Quercetin		rvH1N1	IC_50_ = 2 µM	
**34**		Herbacetin		rvH1N1	IC_50_ = 9 µM	
**35**		Gossypetin		rvH1N1	IC_50_ = 3 µM	
**36**		Cosmosiin		rvH1N1	IC_50_ = 47 µM	
**37**		Astragalin		rvH1N1	IC_50_ = 38 µM	
**38**		Rhodiolinin		rvH1N1	IC_50_ = 10 µM	
**39**		Rhodionin		rvH1N1	IC_50_ = 32 µM	
**40**		Rhodiosin		rvH1N1	IC_50_ = 57 µM	
**41**		Linocinamarin		rvH1N1	IC_50_ = 44 µM	
**42**		Rutin		rvH1N1	IC_50_ = 34 µM	
**43**		Nicotiflorin		rvH1N1	IC_50_ = 32 µM	
**44**	Elderberries	5,7,3′,4′-tetra-O-methylquercetin		H1N1 A/PR/8/34	IC_50_ = 0.4 µM	[62]
**45**		(±)-Dihydromyricetin		H1N1 A/PR/8/34	IC_50_ = 9 µM	
**46**	*Salvia plebeia* R. Br.	Hispidulin		H1N1 A/PR/8/34	IC_50_ = 20 µM	[63]
**47**		Nepetin		H1N1 A/PR/8/34	IC_50_ = 11 µM	
**48**		Rosmarinic acid methyl ester		H1N1 A/PR/8/34	IC_50_ = 17 µM	
**-**	Honeysuckle	Acids-flavonoids		H1N1	EC_50_ = 4 µg/mL	[64]
**49**	*Commelina communis* L.	Homonojirimycin		A/PR/8/34 (H1N1)	EC_50_ = 10 μg/mL	[65]
**50**	*Jishengella endophytica* 161111	Perlolyrine		H1N1 A/PR/8/34	IC_50_ = 38 μg/mL	[66]
**51**		1-hydroxy-β-carboline		H1N1 A/PR/8/34	IC_50_ = 25 μg/mL	
**52**		Lumichrome		H1N1 A/PR/8/34	IC_50_ = 40 μg/mL	
**53**		1H-indole-3-carboxaldehyde		H1N1 A/PR/8/34	IC_50_ = 46 μg/mL	
**54**	Marine sponges *Pericharax heteroraphis*	Leucettamine C		H1N1 A/PR/8/34	33% Inhibition at 50 μg/mL	[67]
**-**	*Peganum harmala* L.	The crude extract		H1N1 A/PR/8/34	IC_50_ = 10 µg/mL	[68]
**-**		Total alkaloid		H1N1 A/PR/8/34	IC_50_ = 6 µg/mL	
**55**	*Papaver somniferum*	Papaverine		A/WSN/33 (H1N1)	IC_50_ = 17 µM	[69]
**56**	*I*. *indigotica* leaves	Isatidifoliumosides	Isatidifoliumosides/Epiisatidifoliumosides C (53/54) in the 3:2 ratio	H1N1 A/PR/8/34	IC_50_ = 65 µmol/L	[70]
**57**		Epiisatidifoliumosides C				
**58**	*Sonneratia paracaseolaris*	Paracaseolins A		H1N1	IC_50_ = 28 µg/mL	[71]
**-**	*Ganoderma lingzhi*	Hot water extract		(A/California/04/2009/(H1N1))	IC_50_ = 15 µg/mL	[72]
**59**	*Trichoderma atroviride* FKI-3849.	Wickerol A		A/PR/8/34 (H1N1)	IC_50_ = 0.1 mg/mL	[73]
**60**		Wickerol B		A/PR/8/34 (H1N1)	IC_50_ = 5 mg/mL	
**61**	*Lemnalia* sp. (No. XSSC201907)	Neolemnane sesquiterpene lineolemnenes F		H1N1	IC_50_ = 6 µM	[74]
**62**	*Celastrus aculeatus* Merr.	Aculeatusane A		A/GZ/GIRD07/09 (H1N1)	IC_50_ = 23 µM	[75]
**63**	*Calotropis gigantea* (Asclepiadaceae)	(+)-pinoresinol 4-O-[6″-O-vanilloyl]- β-D-glucopyranoside		A/PR/8/34 (H1N1)	IC_50_ = 25 µM	[76]
**64**	Tea polyphenols	(-)-epigallocatechin (EGC)		A/PR/8/34 (H1N1)	IC_50_ = 31 µg/mL	[77]
**65**		(-)-epigallocatechingallate (EGCG)		A/PR/8/34 (H1N1)	IC_50_ = 56 µg/mL	
**66**		Procyanidin B-2		A/PR/8/34 (H1N1)	IC_50_ = 51 µg/mL	
**67**		Procyanidin B-23,3′-di-O-gallate		A/PR/8/34 (H1N1)	IC_50_ = 35 µg/mL	
**68**		Theaflavin		A/PR/8/34 (H1N1)	IC_50_ = 16 µg/mL	
**-**	*Gyrodinium impudium*	The sulfated exopolysaccharide, p-KG03		A/PR/8/34 (H1N1)	EC_50_ = 0.5 µg/mL	[78]
**-**				wild-type (WT) H1N1	EC_50_ = 0.2 µg/mL	
**-**	*A. nodosum*	Cold water extract		A/PR/8/34 (H1N1)	IC_50_ = 112 µg/mL	[79]
**-**		Hot water extract		A/PR/8/34 (H1N1)	IC_50_ = 101 µg/mL	
**-**		2% Na_2_CO_3_ extract		A/PR/8/34 (H1N1)	IC_50_ = 152 µg/mL	
**-**		0.5 mol^−1^NaOH extract		A/PR/8/34 (H1N1)	IC_50_ = 191 µg/mL	
**-**	*F. vesiculosus*	Cold water extract		A/PR/8/34 (H1N1)	IC_50_ = 75 µg/mL	
**-**		Hot water extract		A/PR/8/34 (H1N1)	IC_50_ = 181 µg/mL	
**-**		2% Na_2_CO_3_ extract		A/PR/8/34 (H1N1)	IC_50_ = 177 µg/mL	
**-**		0.5 mol^−1^ NaOH extract		A/PR/8/34 (H1N1)	IC_50_ = 125 µg/mL	
**-**	Red algae *Eucheuma denticulatum*	85% ethanol extract		H1N1	IC_50_ = 277 µg/mL	[80]
**-**		Water extract		H1N1	IC_50_ = 366 µg/mL	
**-**		4% NaOH extract		H1N1	IC_50_ = 436 µg/mL	
**69**	Roots of *Ilex asprella*	Asprellcoside A		A/PR/8/34 (H1N1)	EC_50_ = 4 mM	[81]
**70**		3,4,5-trimethoxyphe β-D-5-O-caffeoyl-Apiofuranosyl-(16) -β-D-Glucopyranoside		A/PR/8/34 (H1N1)	EC_50_ = 2 mM	
**71**	*Angelica dahurica*	Isoimperatorin		A/PR/8/34 (H1N1)	EC_50_ = 8 µM	[82]
**72**		Oxypeucedanin		A/PR/8/34 (H1N1)	EC_50_ = 6 µM	
**73**		Oxypeucedanin hydrate		A/PR/8/34 (H1N1)	EC_50_ = 11 µM	
**74**		Imperatorin		A/PR/8/34 (H1N1)	EC_50_ = 11 µM	
**75**	Gentiopicroside derivatives	2′,3′,6′-Tri-O-benzoyl-4′- O-methylsulfonyl gentiopicroside		A/WSN/33 (H1N1)	IC_50_ = 39.5 µM	[83]
**76**	Gentiopicroside derivatives	4′-fluoro-4′-deoxy gentiopicroside		A/WSN/33 (H1N1)	IC_50_ = 45.2 µM	[83]
**77**	Gentiopicroside derivatives	2′,3′,6′-Tri-O-benzoyl-4′,5′-olefin gentiopicroside		A/WSN/33 (H1N1)	IC_50_ = 44.0 µM	[83]
**78**	Triptolide derivatives	4-(((5bS, 6aS, 7aR, 8R, 8aS, 9aS, 9bS, 10aS, 10bS)-8a-isopropyl-10bmethyl-3-oxo 1, 2,3, 5,5b, 6,6a, 8,8a, 9a, 9b, 10b dodecahydrotris(oxireno) [2′, 3′:4b, 5;2″, 3″:6, 7;2‴, 3‴:8a, 9] phenanthro[1, 2-c] furan-8-yl)oxy)-2, 2-dimethyl-4 oxobutanoic acid		A/WSN/33 (H1N1)	EC_50_ = 3.24 µM	[84]
**79**	(-)-Borneol derivatives	N,N,N-Trimethyl-2-oxo-2-((1S,2R,4S)-1,7,7-trimethylbicyclo [2.2.1]heptan-2-yloxy)ethanaminium iodide		A/PR/8/34 (H1N1)	IC_50_= 2.4 μM	[85]
**80**	Andrographolide derivatives	14-alpha-lipoyl andrographolide		A/PR/8/34 (H1N1)	EC_50_ = 7.2 µM	[86]
**81**	Resveratrol derivatives	R42		A/PR/8/34 (H1N1)	IC_50_ = 3.56 µM	[87]
**82**	Pterodontic acid derivatives	Pterodontic acid derivatives compound 15		H1N1	IC_50_ = 9.92 µM	[88]
**83**	Gastrodin derivatives	Methyl 4-fluoro-3-((2S,3R,4S,5R,6R)-3,4,5-triacetoxy-6-(acetoxymethyl)-tetrahydro-2H-pyran-2-yloxy) benzoate		A/FM/1/47(H1N1)	IC_50_ = 34.4 μM	[89]
**84**	Mannich bases of abietic acid derivatives	Mannich bases of abietic acid derivatives compound 12		A/PR/8/34 (H1N1)	IC_50_ = 5.0 μM	[90]
**85**	Terpenophenols and some of their N- and O-derivatives	2-(1,7,7-Trimethylbicyclo[2.2.1]hept-exo-2-yl)cyclohexa-2,5-dien-1,4-dione		A/PR/8/34 (H1N1)	IC_50_ = 0.5 µM	[91]
**86**	Ferulic acid derivatives	(E)-3-(4-Hydroxy-3-methoxyphenyl)-1-(4-methylpiperazin-1-yl)-prop-2-en-1-one		H1N1	IC_50_ = 12.77 ± 0.47 μg/mL	[92]

**Table 2:** Pharmacological and molecular parameters of Anti-H1N1 phytochemicals from natural resource.

No	Name	MW	Alog *P*	Hdon	Hacc	OB (%)	Cao-2	BBB	DL	RBN
**24**	Kaempferol-3-sophoroside	610.57	−2.07	10	16	5.30	−2.42	−3.27	0.71	7
**27**	Kaempferol-3-glucoside	448.41	−0.32	7	11	2.77	−1.36	−1.99	0.74	4
**28**	Quercetin-3-sophoroside	626.57	−2.33	11	17	3.37	−3.39	0.67	0.30	7
**29**	Luteolin	286.25	2.07	4	6	36.16	0.19	−0.84	0.25	1
**32**	Kaempferol	286.25	1.77	4	6	41.88	0.26	−0.55	0.24	1
**33**	Quercetin	302.25	1.50	5	7	46.43	0.05	−0.77	0.28	1
**34**	Herbacetin	302.25	1.50	5	7	36.07	0.12	−0.65	0.27	1
**35**	Gossypetin	318.25	1.24	6	8	35.00	−0.09	−1.02	0.31	1
**36**	Cosmosiin	432.41	0.43	6	10	9.68	−1.08	−2.26	0.74	4
**37**	Astragalin	448.41	−0.32	7	11	14.03	−1.24	−1.97	0.74	4
**42**	Rutin	610.57	−1.45	10	16	3.20	−1.93	−2.75	0.68	6
**43**	Nicotiflorin	594.57	−1.18	9	15	3.64	−1.77	−2.55	0.73	6
**46**	Hispidulin	300.28	2.32	3	6	30.97	0.48	−0.49	0.27	2
**47**	Nepetin	316.28	2.05	4	7	26.75	0.37	−0.78	0.31	2
**50**	Perlolyrine	264.30	3.20	2	3	65.95	0.88	0.15	0.27	2
**53**	1H-indole-3-carboxaldehyde	145.17	1.88	1	1	19.82	1.25	1.17	0.04	1
**55**	Papaverine	339.42	3.50	0	5	64.04	1.22	0.57	0.38	6
**65**	(-)-epigallocatechin	306.29	1.65	6	7	24.18	−0.22	−0.82	0.27	1
**66**	Procyanidin B-2	578.56	3.36	10	12	3.01	−1.14	−2.02	0.66	3
**67**	ProcyanidinB-23,3′-di-O-gallate	902.88	−0.13	16	22	3.01	−3.60	−4.93	0.17	9
**71**	Isoimperatorin	270.30	3.65	0	4	45.46	0.97	0.66	0.23	3
**72**	Oxypeucedanin	286.30	2.00	0	5	24.90	0.85	0.11	0.30	3
**74**	Imperatorin	270.30	3.65	0	4	34.55	1.13	0.92	0.22	3

MW, molecular weight; Alog *P*, lipophilicity; Hdon, hydrogen bond donors; Hacc, hydrogen bond acceptor; OB (%), oral bioavailability; Cao-2, Caco-2 permeability; BBB, blood–brain barrier; DL, drug-likeness; RBN, the number of bonds that allow free rotation around themselves.

## Data Availability

Not applicable.
